# Supplementary material for: Evaluating insect-host interactions as a driver of species divergence in palm flower weevils
Source: Commun Biol. 2020 Dec 9;3:749. doi: 10.1038/s42003-020-01482-3 (PMC7726107; doi:10.1038/s42003-020-01482-3)
Supplement: Supplementary file 1 — Supplementary Information [file 42003_2020_1482_MOESM1_ESM.pdf]

Evaluating insect-host interactions as a driver of population divergence in palm flower weevils

Supplementary Figures and Tables

Bruno A. S. de Medeiros
Smithsonian Tropical Research Institute and
Museum of Comparative Zoology, Harvard University
demedeirosb@si.edu

Brian D. Farrell
Museum of Comparative Zoology, Harvard University
bfarrell@oeb.harvard.edu

Contents

1 Supplementary Figures **2**

2 Supplementary Tables **10**

1 Supplementary Figures

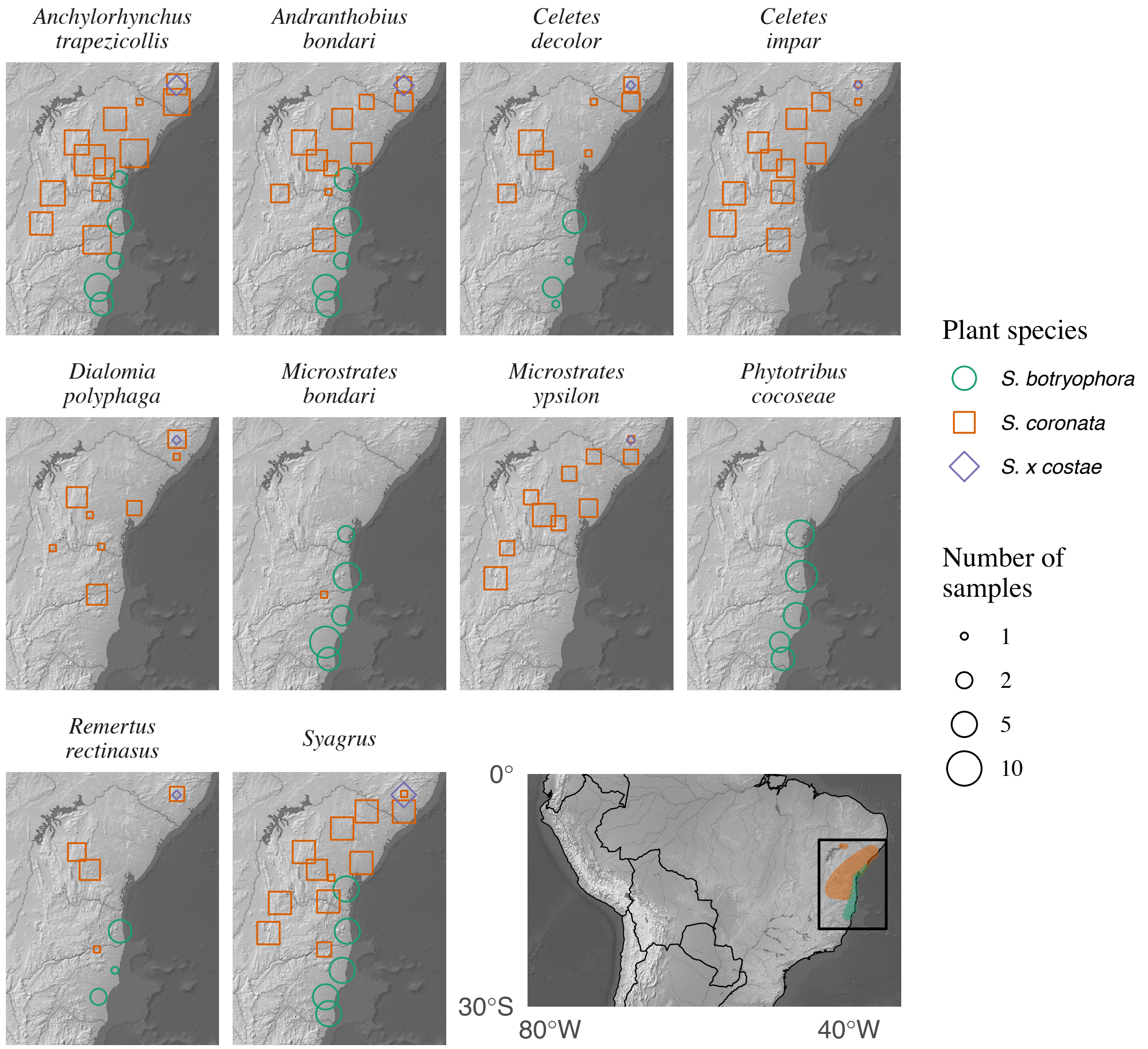

Supplementary Figure 1: Sampling for species of palms and weevils. Large map shows the known distribution of palm species [1, 2]

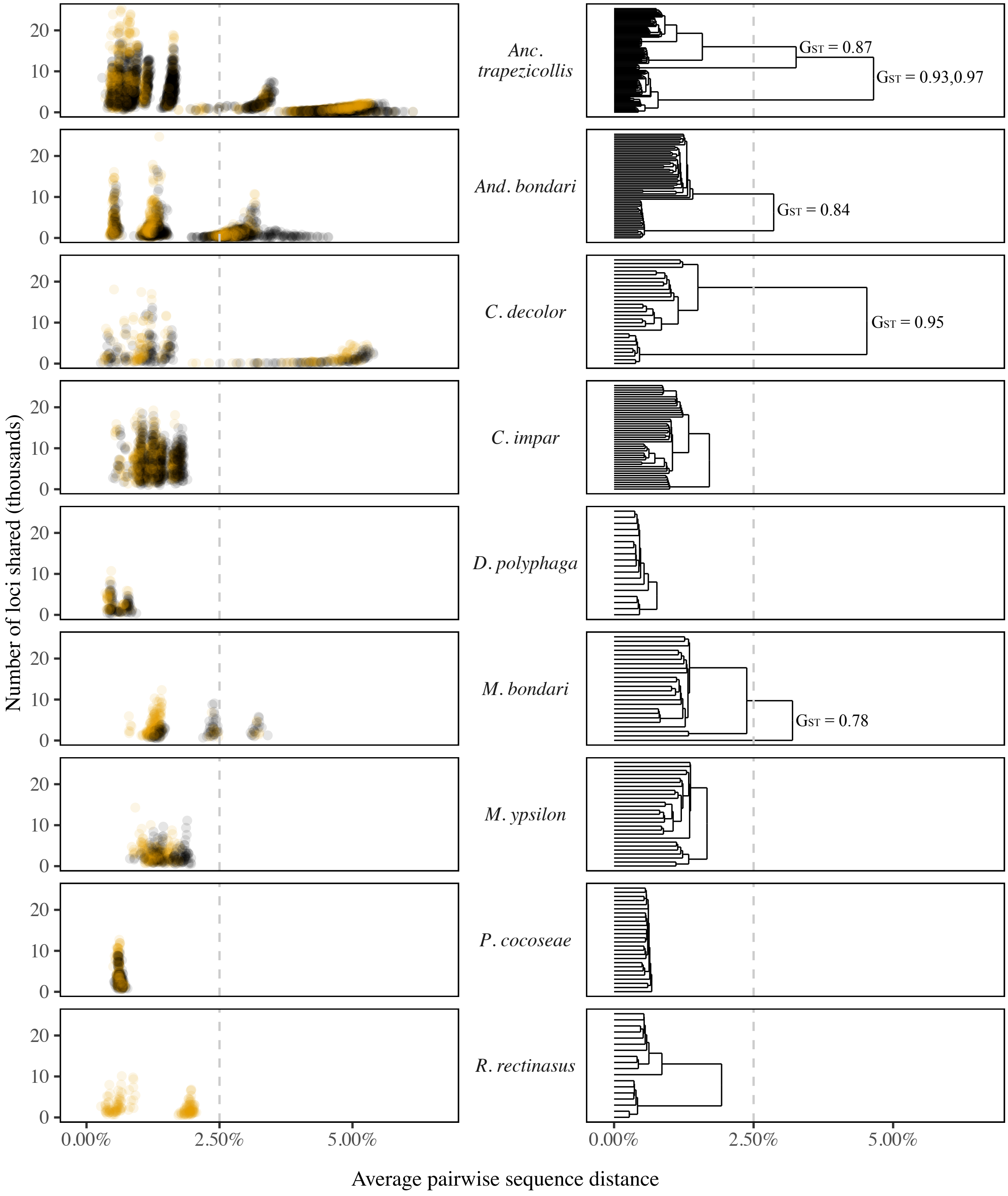

Supplementary Figure 2: Left: pairwise sequence distance against pairwise number of loci shared. Yellow points show samples prepared in the same batch. Right: UPGMA clustering of samples based on pairwise sequence distance. Dashed line separates clusters at 2.5% divergence, with $G\prime_{ST}$ between these clusters shown.

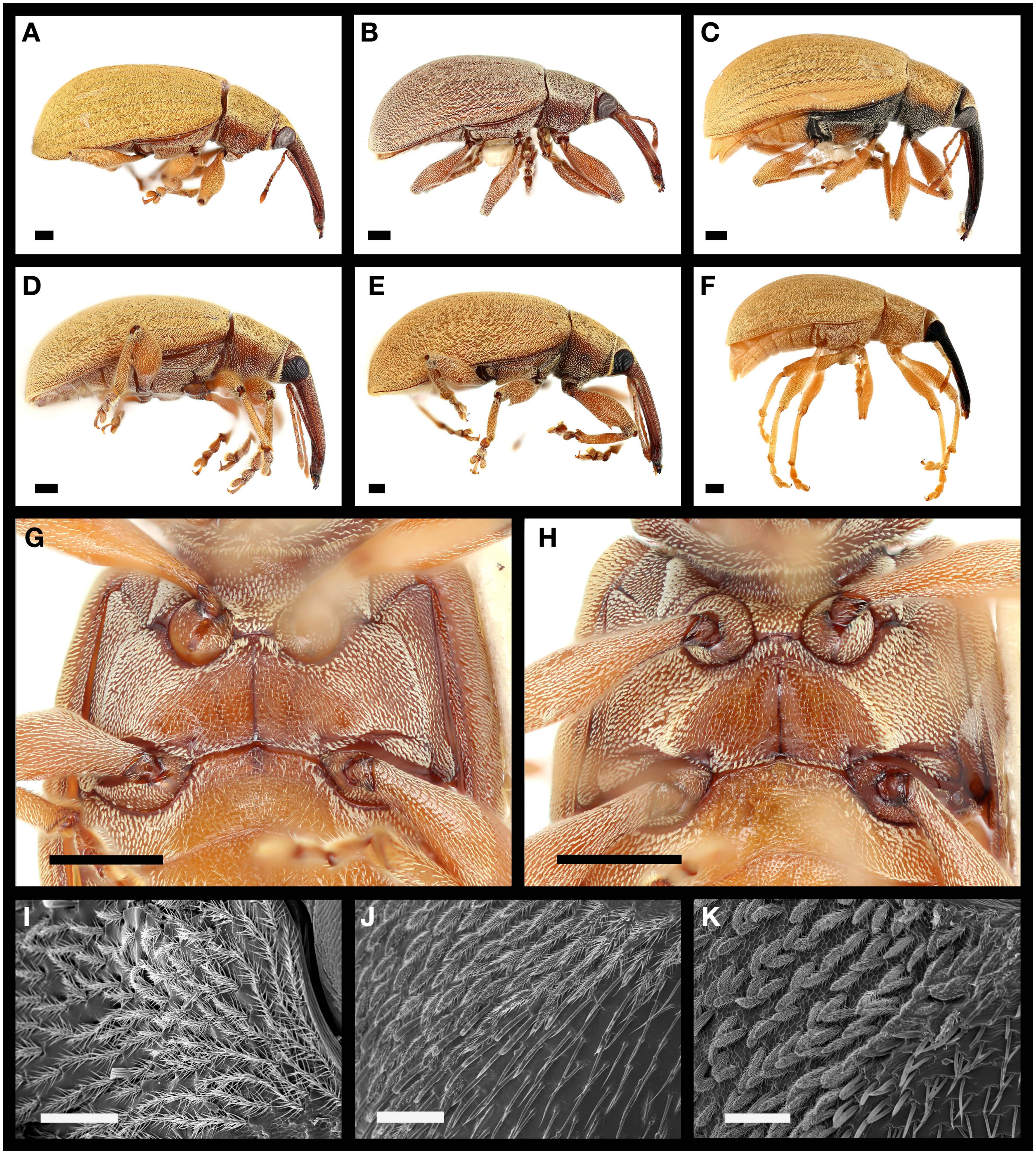

Supplementary Figure 3: Morphology of *Anchylorhynchus trapezicollis* OTU 1 (**A–D,G,I–J**), OTU 2 (**E,H,K**) and OTU 3 (**F**). Black scales: 500 μm, white scales: 100 μm. See Figure 1 in main text for cluster labels. **A**: OTU 1, cluster C, **B**: OTU 1, cluster A (east), **C,G**: OTU 1, cluster A (west). **D**: OTU 1, cluster B. **E,H**: OTU 2, cluster B. Specimens **D–E,G–H** were collected in the same inflorescence. **I**: Ventral plumose setae in OTU 1, cluster B (in *Syagrus botryophora*) are very elongated and branched. **J**: Border of male metasternal concavity in OTU 1, cluster A (in *Syagrus coronata*), showing shorter setae in these populations. **K**: Border of metasternal concavity in OTU 2, cluster B, even shorter and less branched setae in this species.

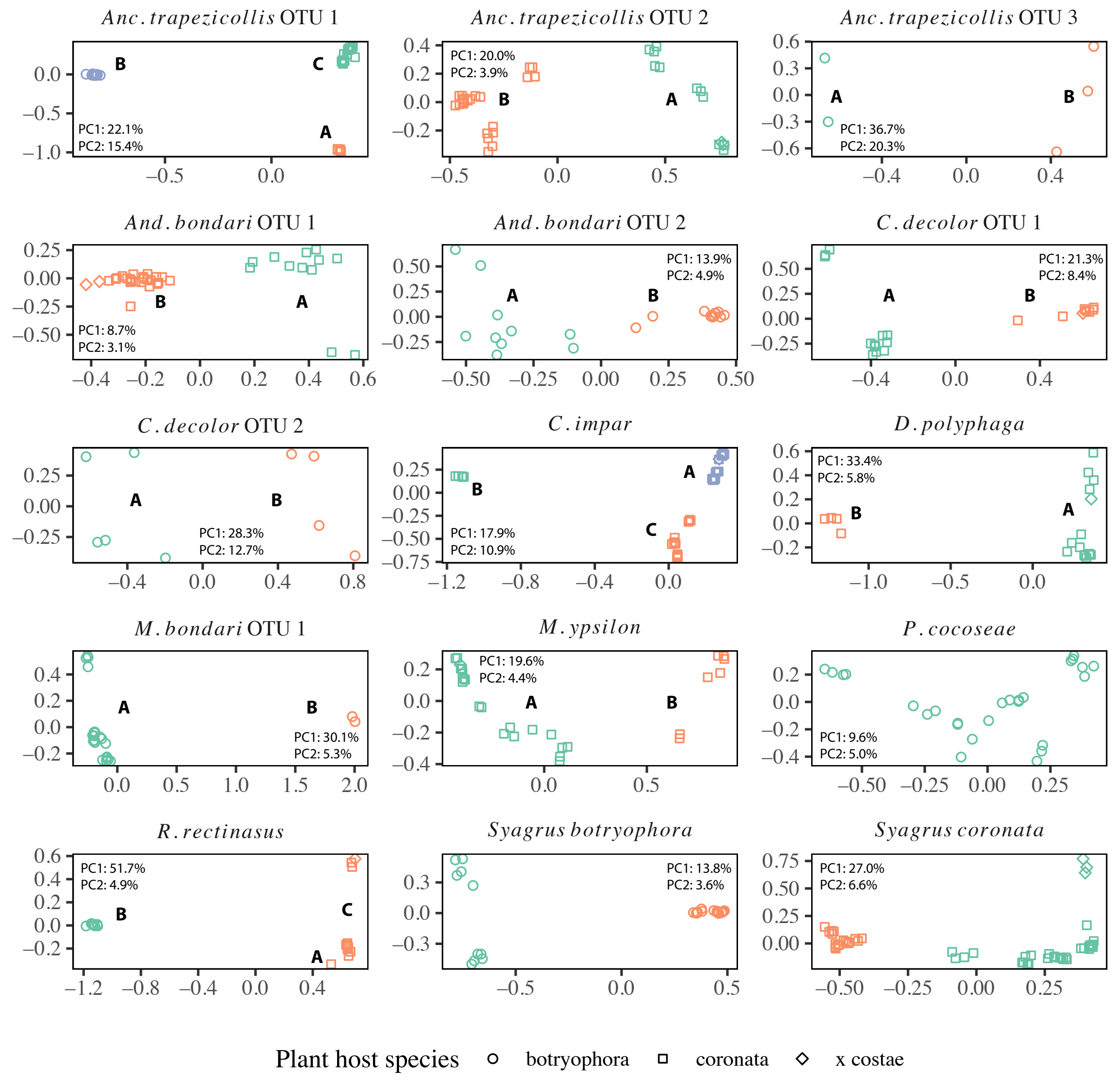

Supplementary Figure 4: Principal component analyses of the genetic variation of weevil OTUs. Independently for each graph, different colors represent different clusters after k-means clustering. Percent variance associated with PC1 and PC2 are provided for each species. Cluster labels (A, B, C) correspond to populations used in coalescent analyses and also to Figure 1 in main text.

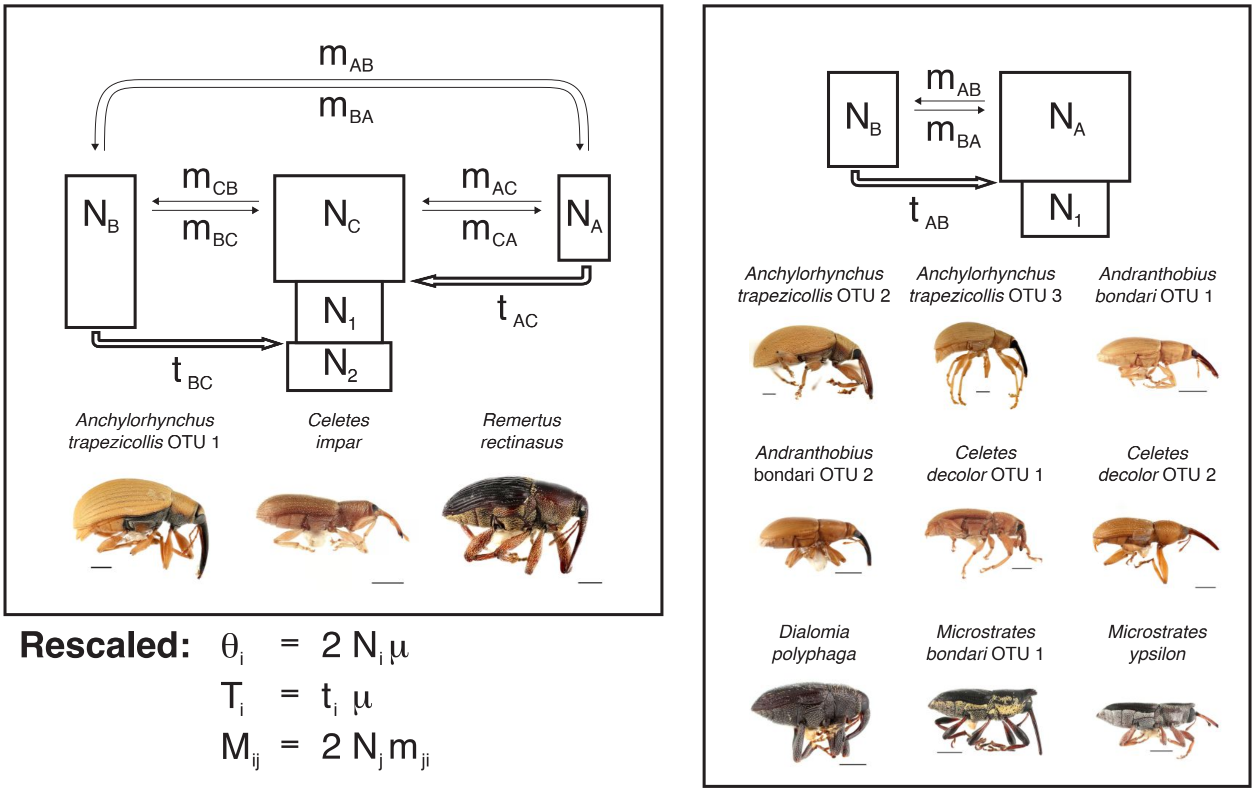

Supplementary Figure 5: Full model of isolation-with-migration for OTUs with 3 or 2 populations. Boxes represent current (A, B, C) and ancestral (1, 2) populations. Thin arrows represent migration and thick arrows past population merge (backwards in time). Parameters inferred with fastsimcoal using a mutation rate (μ) of 3e-9 were: **N** population sizes, **t** time to population merge (backwards in time) in generations, **m** migration rates (backwards in time). Inferred parameters were scaled to be independent of the unknown mutation rate using the equations in the figure. Images of *A. bondari* OTU 1, *C. decolor* OTU 1, *C. impar*, *D. polyphaga*, *M. ypsilon* and *R. rectinasus* OTU 1 reproduced from de Medeiros et al [3] with permission of Oxford University Press and The Linnean Society of London.

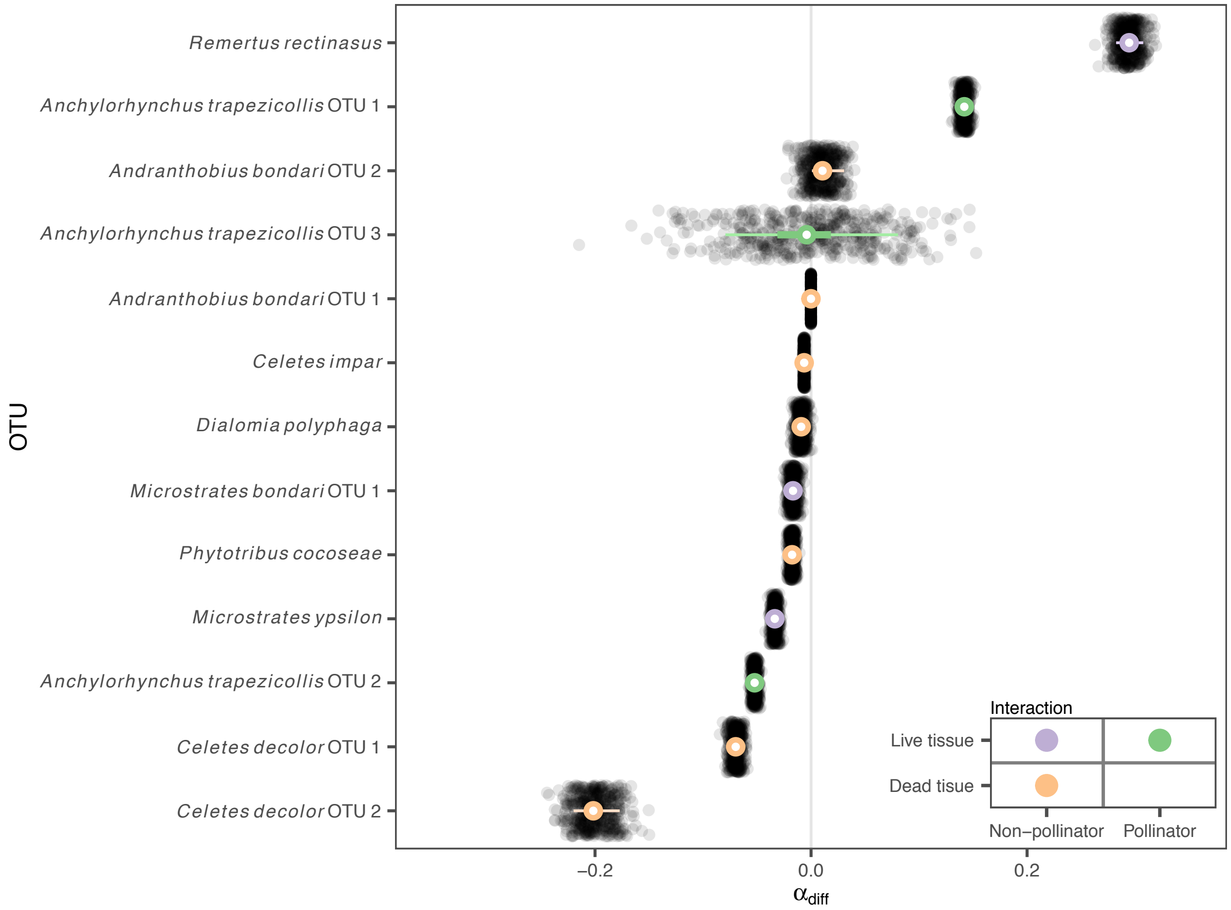

Supplementary Figure 6: Posterior predictive simulations (points) show that the Bayesian hierarchical model has a good fit to the estimates of α_{diff} across species (colored ranges).

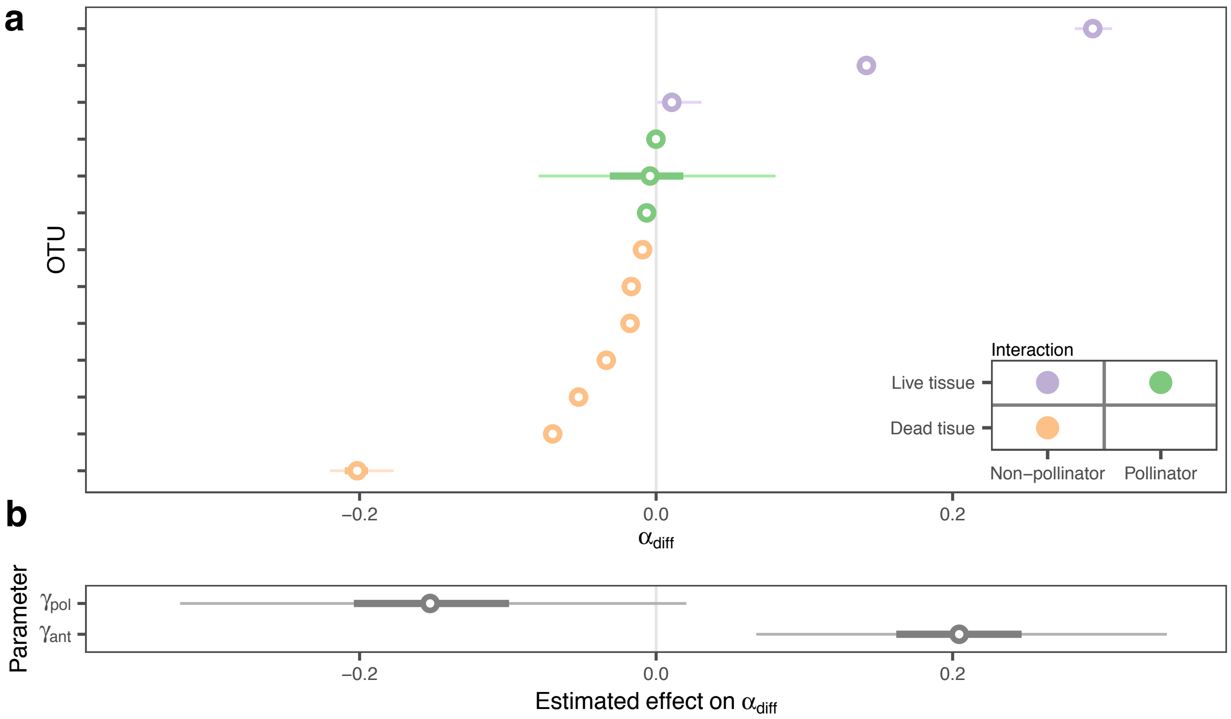

Supplementary Figure 7: Relabelling of OTUs shows that the sample size and input data distribution in this study provide enough power to estimate γ parameters. **a** Relabelled input data used as model input. **b** Estimated γ parameters for this data.

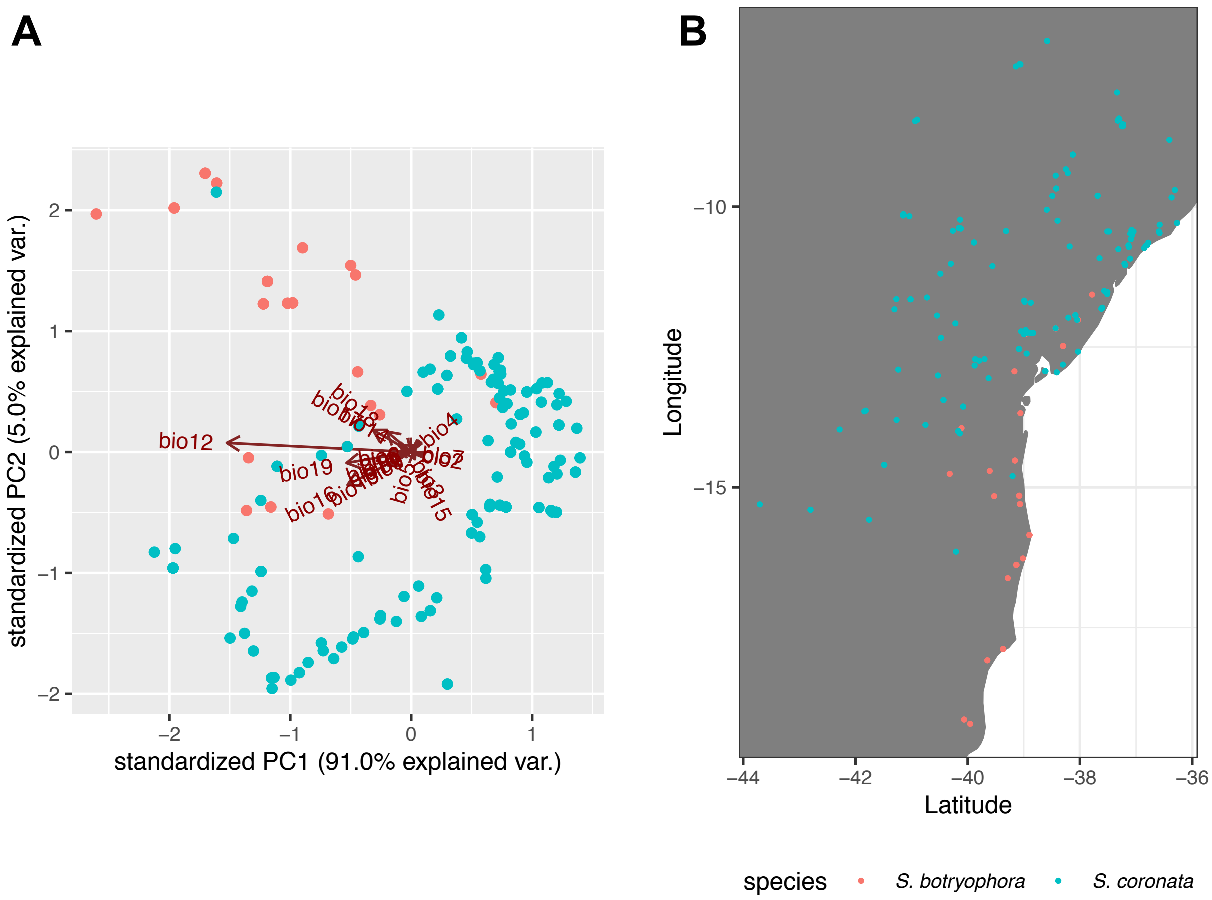

Supplementary Figure 8: Annual precipitation explains most of the variation in climate across the distribution of both palms. **A** Principal component analysis of all bioclimatic variables extracted for occurrences of *Syagrus botryophora* and *Syagrus coronata*, showing the very high loading of bio12 (Annual Precipitation) on PC1, which explains over 90% of the variance . **B** Map of occurences used for this analysis.

2 Supplementary Tables

Supplementary Table 1: Models fitted in fastsimcoal, ordered by AIC scores and including maximum likelihood estimates. See Figure 5 for a visual scheme of model parameters. Preferred model for each OTU has the smallest AIC.

OTU	Model	AIC	ΔAIC	θ_A	θ_B	θ_C	θ_1	θ_2	T_1	T_2	M_{AB}	M_{BA}	M_{AC}	M_{CA}	M_{BC}	M_{CB}
Anchylorhynchus	full	481206	0	3.31E-03	3.69E-03	1.17E-02	2.15E-03	1.39E-03	3.61E-03	8.71E-03	0.07	0.05	0.19	0.49	0.15	0.18
trapezicollis OTU 1	no A,B	483054	1848	3.32E-03	3.59E-03	1.19E-02	2.67E-03	4.00E-03	3.32E-03	6.19E-03			0.39	0.23	0.22	0.21
	no A	485764	4558	3.49E-03	3.51E-03	1.28E-02	5.06E-03	4.06E-03	1.84E-03	5.49E-03					0.23	0.17
	no B,C	486572	5366	3.46E-03	3.85E-03	1.25E-02	1.37E-03	5.56E-03	2.98E-03	3.66E-03	0.08	0.1	0.04	0.88		
	no B	488324	7118	3.32E-03	3.97E-03	1.19E-02	1.23E-03	6.30E-03	2.66E-03	2.98E-03			0.17	0.36		
	no C	488906	7700	3.32E-03	3.70E-03	1.60E-02	3.99E-03	5.80E-03	1.87E-03	3.37E-03	0.03	0.09				
	no mig	489805	8599	3.27E-03	3.62E-03	1.40E-02	2.44E-03	6.35E-03	1.86E-03	2.62E-03						
Anchylorhynchus	full	428578	0	3.32E-03	4.30E-03		7.96E-04		6.45E-03		0.99	1.15				
trapezicollis OTU 2	no mig	434224	5645	3.27E-03	4.65E-03		5.29E-03		5.70E-04							
Anchylorhynchus	full	74284	0	1.42E-01	3.06E-03		1.85E-02		4.00E-03		15252.22	0.27				
trapezicollis OTU 3	no mig	74512	228	3.54E-03	3.24E-03		6.69E-03		3.04E-05							
Andranthobius	full	637902	0	6.62E-03	1.79E-02		3.51E-03		3.70E-03		9.6	21.46				
bondari OTU 1	no mig	644175	6273	2.03E-02	5.23E-02		7.91E-03		5.87E-04							
Andranthobius	full	350204	0	3.47E-03	8.55E-03		1.11E-03		5.35E-03		4.21	9.78				
bondari OTU 2	no mig	353090	2886	5.53E-03	3.65E-02		5.72E-03		3.24E-04							
Celetes	full	359439	0	1.57E-02	3.06E-03		2.67E-03		4.34E-03		1.78	0.88				
decolor OTU 1	no mig	363095	3656	2.94E-02	3.99E-03		5.88E-03		1.26E-03							
Celetes	full	99728	0	3.52E-03	4.66E-03		6.47E-04		9.29E-03		3.37	3.73				
decolor OTU 2	no mig	99910	182	3.69E-03	6.95E-03		5.99E-03		3.19E-04							
Celetes impar	full	644259	0	7.44E-03	3.49E-03	6.66E-03	1.98E-03	3.50E-04	4.33E-03	8.89E-03	0.08	0.13	0.61	2.14	0.38	0.23
	noAC	645339	1080	7.43E-03	3.46E-03	6.58E-03	1.98E-03	4.53E-04	4.33E-03	9.19E-03			1.04	1.97	0.57	0.28
	no A,B	646163	1904	7.89E-03	3.51E-03	6.08E-03	1.07E-03	1.14E-03	6.53E-03	1.95E-03	0.08	0.3	0.53	2.69		
	no A	649068	4809	7.11E-03	3.36E-03	6.12E-03	1.33E-03	2.83E-03	4.79E-03	1.40E-03			1.18	2.24		
	no B,C	650212	5952	7.14E-03	3.48E-03	8.77E-03	1.16E-02	2.06E-03	8.81E-04	5.42E-03	0.14	0.12			0.68	0.41
	no C	651627	7368	7.14E-03	3.46E-03	7.21E-03	1.27E-02	2.93E-03	7.98E-04	4.77E-03					0.9	0.25
	no B	654584	10325	5.94E-03	3.56E-03	9.38E-03	1.25E-02	3.67E-03	7.77E-04	4.17E-03	0.04	0.8				
	no mig	657909	13649	6.96E-03	4.01E-03	9.67E-03	9.76E-03	6.06E-03	9.19E-04	2.17E-03						
Dialomia polyphaga	full	107341	0	4.30E-03	3.39E-03		8.61E-04		5.18E-03		0.34	0.59				
	no mig	108242	902	5.89E-03	3.06E-03		1.83E-03		1.32E-03							
Microstrates	full	135833	0	1.47E-02	3.46E-03		3.60E-04		8.04E-03		0.17	0.24				
bondari OTU 1	no mig	136929	1096	1.68E-02	3.76E-03		1.09E-03		3.63E-03							
Microstrates ypsilon	full	218442	0	1.06E-02	4.48E-03		1.67E-03		5.64E-03		1.27	1.06				
	no mig	221062	2620	1.34E-02	5.77E-03		5.98E-03		1.25E-03							
Remertus rectinasus	no B,C	112239	0	3.64E-03	3.24E-03	3.75E-03	4.72E-04	1.11E-04	2.96E-03	1.14E-02	0.31	0.03	3.82	1.57		
	full	112254	15	6.12E-03	3.46E-03	4.42E-03	5.16E-04	8.86E-05	2.65E-03	1.10E-02	0.49	0	8.03	0.39	0.02	0.07
	no A,B	112307	68	7.66E-03	3.50E-03	3.70E-03	6.36E-04	6.90E-05	1.09E-02	1.90E-03			12.01	0	0.08	0.04
	noAC	112372	134	1.03E-02	3.24E-03	5.36E-03	1.20E-03	3.93E-04	1.04E-03	1.04E-02	0.61	0			0.06	0.11
	no B	112878	639	7.25E-03	3.51E-03	3.18E-03	9.98E-04	3.73E-04	1.07E-02	1.07E-03			18.81	0.47		
	no A	113038	800	2.57E-02	3.46E-03	4.37E-03	4.62E-04	8.64E-05	1.31E-03	1.08E-02					0.08	0.07
	no C	113224	986	4.76E-03	3.24E-03	6.60E-03	4.11E-03	7.35E-03	5.86E-04	5.97E-03	0.41	0.1				
	no mig	113631	1392	6.84E-03	3.06E-03	5.05E-03	1.79E-03	5.05E-03	1.04E-03	4.26E-03						

Supplementary Table 2: Model choice by cross validation in BEDASSLE2. For each OTU, models are ordered by average predictive value. The preferred model (in bold) is the one including fewer parameters among those with highest posterior predictive value, considering the 95% confidence intervals. Models are labelled after the distance matrices included: geographical distance (*g*), plant host genetic distance (*p*) and climatic distance (*c*). In the null model, genetic covariance across samples does not depend deterministically on any other distance matrix.

OTU	Model	Predictive value, mean (95% confidence interval)
Anchylorhynchus trapezicollis OTU 1	**gp**	**81987.80(81987.69–81987.92)**
	gpc	81987.52(81987.42–81987.63)
	pc	81982.12(81982.02–81982.22)
	p	81982.07(81981.97–81982.17)
	gc	80228.69(80228.58–80228.80)
	c	78185.06(78184.95–78185.18)
	g	77827.47(77827.39–77827.55)
	null	69095.10(69095.01–69095.20)
Anchylorhynchus trapezicollis OTU 2	gp	50557.87(50557.77–50557.97)
	gpc	50557.84(50557.77–50557.92)
	**g**	**50557.78(50557.71–50557.86)**
	gc	50557.75(50557.67–50557.83)
	pc	49903.85(49903.76–49903.94)
	p	49903.74(49903.65–49903.83)
	c	49058.37(49058.28–49058.46)
	null	46977.82(46977.73–46977.91)
Anchylorhynchus trapezicollis OTU 3	**gp**	**1467.62(1467.60–1467.64)**
	gpc	1467.58(1467.56–1467.60)
	p	1467.40(1467.38–1467.42)
	g	1467.33(1467.31–1467.35)
	pc	1467.26(1467.24–1467.28)
	gc	1467.15(1467.14–1467.17)
	c	1466.17(1466.15–1466.18)
	null	1458.93(1458.92–1458.95)
Andranthobius bondari OTU 1	**g**	**70595.05(70594.95–70595.14)**
	gp	70595.02(70594.93–70595.12)
	gc	70595.00(70594.91–70595.09)
	gpc	70594.67(70594.58–70594.75)
	p	70581.85(70581.76–70581.95)
	pc	70581.83(70581.75–70581.90)
	c	70579.11(70579.02–70579.21)
	null	70571.69(70571.59–70571.78)
Andranthobius bondari OTU 2	**p**	**29694.04(29693.95–29694.12)**
	pc	29693.86(29693.78–29693.94)
	gp	29693.75(29693.68–29693.82)
	gpc	29693.72(29693.65–29693.79)
	g	29690.56(29690.49–29690.63)
	c	29690.13(29690.05–29690.20)
	gc	29688.93(29688.87–29688.99)
	null	29646.84(29646.76–29646.93)
Celetes decolor OTU 1	**g**	**19118.70(19118.64–19118.76)**
	gc	19118.70(19118.65–19118.74)
	gp	19118.67(19118.62–19118.72)
	gpc	19118.49(19118.44–19118.54)
	p	18925.93(18925.87–18925.98)
	pc	18916.29(18914.95–18917.63)
	c	18751.01(18750.95–18751.06)
	null	18030.67(18030.61–18030.73)

Supplementary Table 2: (continued)

Species	Model	Predictive value, mean (95% confidence interval)
Celetes decolor OTU 2	**g**	**3423.65(3423.63–3423.68)**
	gp	3423.55(3423.53–3423.58)
	gpc	3423.51(3423.48–3423.55)
	gc	3423.28(3423.21–3423.35)
	c	3409.73(3409.59–3409.88)
	pc	3408.21(3407.67–3408.75)
	p	3392.80(3392.76–3392.84)
	null	3299.74(3299.71–3299.76)
Celetes impar	gpc	87977.06(87976.94–87977.18)
	**gp**	**87977.02(87976.92–87977.13)**
	gc	87973.52(87973.40–87973.64)
	g	87973.40(87973.28–87973.51)
	pc	87621.84(87621.74–87621.95)
	p	87621.82(87621.72–87621.91)
	c	86979.59(86979.48–86979.70)
	null	83617.98(83617.87–83618.09)
Dialomia polyphaga	gc	7679.66(7679.63–7679.70)
	**g**	**7679.64(7679.60–7679.67)**
	gp	7679.61(7679.57–7679.64)
	gpc	7679.50(7679.47–7679.53)
	p	7632.07(7632.00–7632.14)
	pc	7631.79(7631.72–7631.86)
	c	7621.51(7621.35–7621.66)
	null	7579.66(7579.63–7579.70)
Microstrates bondari OTU 1	**gp**	**13190.15(13190.07–13190.22)**
	gpc	13189.95(13189.89–13190.01)
	g	13182.32(13182.28–13182.36)
	gc	13182.28(13182.24–13182.33)
	pc	13177.53(13177.49–13177.58)
	p	13172.54(13172.50–13172.59)
	c	13145.76(13145.61–13145.92)
	null	13102.05(13102.01–13102.10)
Microstrates ypsilon	**gp**	**18338.82(18338.77–18338.87)**
	gpc	18338.76(18338.72–18338.81)
	g	18337.02(18336.97–18337.07)
	gc	18336.90(18336.85–18336.96)
	p	18253.96(18253.92–18254.00)
	pc	18253.94(18253.88–18253.99)
	c	18051.73(18051.67–18051.78)
	null	17820.16(17820.10–17820.21)
Phytotribus cocoseae	**gp**	**22587.74(22587.67–22587.81)**
	gpc	22587.68(22587.61–22587.75)
	g	22584.24(22584.17–22584.30)
	gc	22584.21(22584.15–22584.27)
	pc	22569.90(22569.84–22569.96)
	c	22563.24(22563.18–22563.30)
	p	22537.11(22537.00–22537.21)
	null	22417.75(22417.69–22417.80)
Remertus rectinasus	**gp**	**10848.63(10848.59–10848.67)**
	gpc	10848.60(10848.56–10848.64)
	p	10843.44(10843.40–10843.48)
	pc	10843.41(10843.36–10843.45)
	gc	10393.50(10393.46–10393.53)
	c	10167.43(10167.40–10167.47)
	g	9933.43(9933.39–9933.47)
	null	7643.13(7640.30–7645.96)

Supplementary Table 3: Mean and 95% credibility intervals for parameters estimated by the best BEDASSLE 2 model for each weevil OTU. α_2 controls the shape of genetic covariance decay, γ is the average covariance across samples and α_0 is the covariance at distance 0. The other α parameters control the contribution of geography or plant host.

plant host	Interaction	species	α_{geo}	α_{plant}	α_0	α_2
S. botryophora	pollinator,antagonist	*Anc. trapezicollis* OTU 3	1.62e (0.43–2.49)e-01	1.58 (0.45–2.43)e-01	0.06 (0.06–0.07)	1.04 (0.36–1.87)
	commensal	*Andranthobius bondari* OTU 2	0.78 (0.02–2.94)e-03	1.14 (0.08–3.33)e-02	0.18 (0.18–0.19)	1.20 (0.72–1.80)
	commensal	*Celetes decolor* OTU 2	2.14 (2.01–2.26)e-01	1.28 (0.06–3.49)e-02	0.14 (0.13–0.15)	1.93 (1.76–2.00)
	commensal	*Phytotribus cocoseae*	4.40 (3.19–5.58)e-02	2.64 (1.49–4.01)e-02	0.18 (0.18–0.19)	1.67 (1.41–1.95)
	antagonist	*Microstrates bondari* OTU 1	5.52 (4.41–6.20)e-02	3.85 (2.88–4.40)e-02	0.20 (0.19–0.21)	1.90 (1.69–2.00)
S. coronata	pollinator,antagonist	*Anc. trapezicollis* OTU 2	5.25 (4.93–5.54)e-02	1.95 (0.06–5.36)e-04	0.20 (0.19–0.20)	1.03 (1.01–1.05)
	commensal	*Andranthobius bondari* OTU 1	0.92 (0.22–2.37)e-04	1.50 (0.03–6.54)e-06	0.22 (0.22–0.23)	0.74 (0.64–0.85)
	commensal	*Celetes decolor* OTU 1	7.01 (6.46–7.57)e-02	0.36 (0.01–1.04)e-03	0.20 (0.19–0.20)	1.13 (1.09–1.17)
	commensal	*Celetes impar*	6.64 (5.90–7.47)e-03	2.54 (1.91–3.18)e-04	0.22 (0.21–0.23)	0.66 (0.64–0.67)
	commensal	*Dialomia polyphaga*	0.92 (0.48–1.54)e-02	0.42 (0.01–1.42)e-04	0.20 (0.19–0.21)	0.43 (0.38–0.50)
	antagonist	*Microstrates ypsilon*	3.92 (3.52–4.32)e-02	5.57 (4.12–7.09)e-03	0.21 (0.20–0.22)	1.32 (1.27–1.37)
both	pollinator,antagonist	*Anc. trapezicollis* OTU 1	1.96 (1.74–2.17)e-02	1.61 (1.57–1.66)e-01	0.23 (0.22–0.23)	1.76 (1.73–1.78)
	antagonist	*Remertus rectinasus*	6.40 (5.70–7.06)e-02	3.59 (3.50–3.67)e-01)	0.20 (0.19–0.21)	2.00 (2.00–2.00)

Supplementary Table 4: Number of samples, populations and SNPs sequenced for each plant species and weevil OTU, following filtering for HWE equilibrium. For weevil OTUs, we also include the number of unlinked SNPs used in BEDASSLE, filtered as described in the main text methods.

Species/OTU	Samples	Populations	All SNPs	variable RAD loci	Unlinked SNPs
Syagrus botryophora	25	5	12532	5300	–
Syagrus coronata	49	13	30089	10081	–
Anchylorhynchus trapezicollis OTU 1	47	13	49408	12829	8607
Anchylorhynchus trapezicollis OTU 2	36	10	38022	12558	8520
Anchylorhynchus trapezicollis OTU 3	5	3	7799	4097	3008
Andranthobius bondari OTU 1	38	12	51948	11724	9098
Andranthobius bondari OTU 2	22	5	32082	11614	8798
Celetes decolor OTU 1	20	8	31706	9505	6206
Celetes decolor OTU 2	9	4	9596	4321	3286
Celetes impar	47	13	56309	11628	9786
Dialomia polyphaga	18	9	10542	4511	2794
Microstrates bondari OTU 1	23	5	15973	3984	3475
Microstrates ypsilon	27	11	21256	5345	3838
Phytotribus cocoseae	26	5	15305	6017	5695
Remertus rectinasus OTU 1	18	8	14371	5006	3694

References

1. Noblick, L. R. A revision of the genus Syagrus (Arecaceae). *Phytotaxa* **294,** 001–262 (2017).
2. GBIF.org. *GBIF Ocurrence Download* 2019. https://doi.org/10.15468/dl.lprfwo.
3. De Medeiros, B. A. S., Núñez-Avellaneda, L. A., Hernandez, A. M. & Farrell, B. D. Flower visitors of the licuri palm (Syagrus coronata): brood pollinators coexist with a diverse community of antagonists and mutualists. *Biological Journal of the Linnean Society* **126,** 666–687 (Mar. 2019).
