# Supplementary material for: Evaluating insect-host interactions as a driver of species divergence in palm flower weevils
Source: Commun Biol. 2020 Dec 9;3:749. doi: 10.1038/s42003-020-01482-3 (PMC7726107; doi:10.1038/s42003-020-01482-3)
Supplement: Supplementary file 2 — Description of Additional Supplementary Files [file 42003_2020_1482_MOESM2_ESM.pdf]

Description of Additional Supplementary Files

File Name: Supplementary Data 1
Description: Zip-compressed folder with text files in csv format including tables with underlaying data for Figures 1–3 and Supplementary Figures 1,2,4,6–8
