# Supplementary material for: Evaluating insect-host interactions as a driver of species divergence in palm flower weevils
Source: Commun Biol. 2020 Dec 9;3:749. doi: 10.1038/s42003-020-01482-3 (PMC7726107; doi:10.1038/s42003-020-01482-3)
Supplement: Supplementary file 5 — Reporting Summary [file 42003_2020_1482_MOESM5_ESM.pdf]

nature research

Corresponding author(s): Bruno A. S. de Medeiros

Last updated by author(s): Oct 17, 2020

Reporting Summary

Nature Research wishes to improve the reproducibility of the work that we publish. This form provides structure for consistency and transparency in reporting. For further information on Nature Research policies, see our Editorial Policies and the Editorial Policy Checklist.

Statistics

For all statistical analyses, confirm that the following items are present in the figure legend, table legend, main text, or Methods section.

n/a	Confirmed	
☐	☒	The exact sample size (*n*) for each experimental group/condition, given as a discrete number and unit of measurement
☐	☒	A statement on whether measurements were taken from distinct samples or whether the same sample was measured repeatedly
☐	☒	The statistical test(s) used AND whether they are one- or two-sided<br>*Only common tests should be described solely by name; describe more complex techniques in the Methods section.*
☐	☒	A description of all covariates tested
☐	☒	A description of any assumptions or corrections, such as tests of normality and adjustment for multiple comparisons
☐	☒	A full description of the statistical parameters including central tendency (e.g. means) or other basic estimates (e.g. regression coefficient) AND variation (e.g. standard deviation) or associated estimates of uncertainty (e.g. confidence intervals)
☐	☒	For null hypothesis testing, the test statistic (e.g. *F*, *t*, *r*) with confidence intervals, effect sizes, degrees of freedom and *P* value noted<br>*Give P values as exact values whenever suitable.*
☐	☒	For Bayesian analysis, information on the choice of priors and Markov chain Monte Carlo settings
☐	☒	For hierarchical and complex designs, identification of the appropriate level for tests and full reporting of outcomes
☐	☒	Estimates of effect sizes (e.g. Cohen's *d*, Pearson's *r*), indicating how they were calculated

Our web collection on statistics for biologists contains articles on many of the points above.

Software and code

Policy information about availability of computer code

Data collection	All software used for data collection is described in the main text, with versions specified. Computer code written for this project is made available in the github repository http://github.com/brunoasm/rad_palm_weevil
Data analysis	All software used for data analysis is described in the main text, with versions specified. Computer code written for this project is made available in the github repository http://github.com/brunoasm/rad_palm_weevil

For manuscripts utilizing custom algorithms or software that are central to the research but not yet described in published literature, software must be made available to editors and reviewers. We strongly encourage code deposition in a community repository (e.g. GitHub). See the Nature Research guidelines for submitting code & software for further information.

Data

Policy information about availability of data

All manuscripts must include a data availability statement. This statement should provide the following information, where applicable:

- Accession codes, unique identifiers, or web links for publicly available datasets
- A list of figures that have associated raw data
- A description of any restrictions on data availability

Demultiplexed Illumina raw reads are deposited in the NCBI SRA, BioProject PRJNA397912, accessions SRR12602029–SRR12602364 for insect samples and SRR12603820–SRR12603892 for plant samples. Source data underlying graphs (Figures 1–3, Supplementary Figures 1,2,4,6–8) is available as Supplementary Data 1.

Field-specific reporting

Please select the one below that is the best fit for your research. If you are not sure, read the appropriate sections before making your selection.

☐ Life sciences ☐ Behavioural & social sciences ☒ Ecological, evolutionary & environmental sciences

For a reference copy of the document with all sections, see nature.com/documents/nr-reporting-summary-flat.pdf

Ecological, evolutionary & environmental sciences study design

All studies must disclose on these points even when the disclosure is negative.

Study description	Tissue samples of 2 species of palm and 9 associated beetle species visiting their flowers were collected across the geographical range of the species. Both insects and plants were sequenced by using double-digest RADseq. We used this data to delimit weevil species and then to fit models of isolation by environment, comparing results for the different insect species while considering the different kinds of interaction with host plants.
Research sample	Individuals of the following species: Arecaceae: Syagrus coronata and Syagrus botryophora; Coleoptera, Curculionidae: Anchylorhynchus trapezicollis, Andranthobius bondari, Celetes decolor, Celetes impar, Dialomia polyphaga, Microstrates bondari, Microstrates ypsilon, Phytotribus platyrhinus, Remertus rectinasus.
Sampling strategy	Samples were obtained from fieldwork in natural populations in 18 different localities across the known range of the palm species.
Data collection	In each locality, insects visiting flowers were sampled by bagging a whole inflorescence and preserved in ethanol. In the lab, they were sorted to morphospecies and identified using the available literature. Plant tissues were obtained from leaf samples from the palm in which insects were sampled and other individuals in the population. In the lab we randomly selected up to 8 individuals per locality for DNA extraction and sequenced them by using double-digest RADseq. Additional individuals were sequenced after identification of cryptic species.
Timing and spatial scale	Collections were done between September and November (i. e. spring) of 2013 and 2014 across a few thousand kilometers throughout the range of both palm species.
Data exclusions	Samples that resulted in very few sequencing reads were excluded from analysis. We also excluded insect species that were only observed in a few populations or for which there was little natural history data available by the time we did DNA sequencing.
Reproducibility	A few samples were sequenced multiple times and in a previous manuscript we showed that they genotype calls were nearly identical (de Medeiros & Farrell 2018).
Randomization	When more than 8 individuals for a species were available for sequencing for a given locality, we chose 10 individuals irrespective of their morphology. After finding out that the dataset included cryptic sympatric species, we sequenced new individuals choosing some that corresponded morphologically to the different putative species. We also randomized the position of samples in DNA extraction plates to avoid potential biases arising from cross-contamination when performing high-throughput automated DNA extractions.
Blinding	After DNA extraction, samples were given numeric codes independent of their locality or species identity. Library preparation, sequencing and the initial steps in bioinformatics were done by referring to these codes only. In the case of cryptic species, we only scored morphological features after separating clusters based on the genetic data alone.

Did the study involve field work? ☒ Yes ☐ No

Field work, collection and transport

Field conditions	Fieldwork was done over a total of 4 months in 4 different trips. This was the onset of the rainy season across most of the region, and specific weather conditions varied.
Location	Northeastern Brazil, in seasonally dry forest and coastal rainforest, between the states of Espírito Santo and Pernambuco.
Access & import/export	Field sampling was done with SISBIO permit #39704-7 from Instituto Chico Mendes de Preservação da Biodiversidade, Brazil. Samples were deposited in collections in Brazil (Herbarium of the Institute of Biosciences, University of São Paulo and Museum of Zoology, University of São Paulo) and exported to the USA as loans.
Disturbance	The study involved bagging and shaking or excising inflorescences of Syagrus coronata and Syagrus botryophora, as well as pieces of folioles or whole leaves for samples deposited in herbaria. Both species have large geographical ranges and dense populations, producing several inflorescences per year. The disturbance, therefore, was minor. Climbing did not involve any piercing equipment that could damage plants.

Reporting for specific materials, systems and methods

We require information from authors about some types of materials, experimental systems and methods used in many studies. Here, indicate whether each material, system or method listed is relevant to your study. If you are not sure if a list item applies to your research, read the appropriate section before selecting a response.

Materials & experimental systems

n/a	Involved in the study
☒	☐ Antibodies
☒	☐ Eukaryotic cell lines
☒	☐ Palaeontology and archaeology
☒	☐ Animals and other organisms
☒	☐ Human research participants
☒	☐ Clinical data
☒	☐ Dual use research of concern

Methods

n/a	Involved in the study
☒	☐ ChIP-seq
☒	☐ Flow cytometry
☒	☐ MRI-based neuroimaging
